# Immunotherapy with or without low-intensity chemotherapy versus conventional chemotherapy as first-line treatment for newly diagnosed B-ALL patients fit for intensive chemotherapy: a propensity score-matched study

**DOI:** 10.3389/fmed.2026.1843800

**Published:** 2026-06-18

**Authors:** Bingqian Huang, Yawen Wang, Yubin Li, Jiahui Mao, Ruixue Wang, Gaoyu Lv, Ming Hou, Yan Shi

**Affiliations:** 1 Department of Hematology, Qilu Hospital of Shandong University, Jinan, Shandong, China; 2 Department of Hematology, The First Affiliated Hospital of Shandong First Medical University, Jinan, Shandong, China

**Keywords:** blinatumomab, conventional chemotherapy, induction therapy, inotuzumab ozogamicin, newly diagnosed B-ALL

## Abstract

**Purpose:**

For B-cell acute lymphoblastic leukemia (B-ALL), the dominance of traditional chemotherapy is being rewritten, with multiple promising novel treatment approaches continually emerging. Immunotherapy (blinatumomab, Blina or inotuzumab ozogamicin, InO) has demonstrated efficacy in the induction phase for elderly patients newly diagnosed with B-ALL, but its role in newly diagnosed patients fit for intensive chemotherapy is being explored. This study aims to evaluate the efficacy and safety of immunotherapy with or without low-intensity chemotherapy in fit patients newly diagnosed with B-ALL.

**Patients and methods:**

We retrospectively enrolled 133 patients with newly diagnosed B-ALL who met the inclusion criteria at Qilu Hospital of Shandong University. Patients were divided into two groups based on whether Blina/InO was used in the first-line induction therapy regimen. The immunotherapy group (IG) consisted of 25 patients, while the chemotherapy group (CG) consisted of 108 patients. Propensity score matching (PSM) was performed to match the IG and CG patients in a 1:1 ratio based on age, sex, risk stratification, comorbidities and Philadelphia chromosome status. After matching, there were 25 patients in each group. Primary research objectives included remission rates and minimal residual disease (MRD) negativity rate. Secondary objectives included adverse events. Data management and statistical analysis were performed using SPSS and R software.

**Results:**

The IG achieved a higher MRD negativity rate (88% vs. 48%, *p* = 0.002). Compared with the CG, the IG had a higher median minimum of neutrophil count (*p* < 0.001) and a higher median minimum of platelet count (*p* = 0.001). Furthermore, the duration of neutrophil count < 0.5 × 10^9^/L (*p* = 0.033), hemoglobin level < 80 g/L (*p* = 0.038) and platelet count < 30 × 10^9^/L (*p* = 0.006) was shorter in the IG. They also experienced fewer pulmonary infections (44% vs. 84%, *p* = 0.003). Additionally, the IG required fewer red blood cell transfusions (4 vs. 8 u, *p* = 0.012) and platelet transfusions (0 vs. 48 u, *p* < 0.001).

**Conclusion:**

In the PSM-based retrospective cohort study, an immunotherapy-based first-line treatment strategy showed promising early treatment responses and tolerability to hematologic toxicity in fit patients newly diagnosed with B-ALL. These exploratory findings provide additional real-world evidence.

## 1 Introduction

B-cell acute lymphoblastic leukemia is a malignant neoplastic disease originating from B-cell lymphoid progenitor cells, accounting for 20% of adult acute leukemia (1–3). Standard chemotherapy regimens achieve complete remission (CR) rates of 70–90% in newly diagnosed adult patients with B-ALL (4, 5). However, they often result in severe bone marrow suppression and a high incidence of adverse reactions due to the narrow therapeutic window and poor selectivity (6, 7). Some patients die during the induction phase due to severe infection or profound thrombocytopenia (8). A study by Bourlon C et al. indicates that the overall induction mortality rate in adult and adolescent patients is approximately 12%, with infection-related complications accounting for 77.8% of early deaths and bleeding events accounting for 16.7% (9). Some patients achieve CR but experience relapse within a short period because severe infection compromises subsequent intensive consolidation therapy (10). Additionally, approximately 30–60% of patients who achieve CR following standard induction chemotherapy remain MRD-positive, a status closely associated with relapse (11–14). As a result, first-line treatment for adult B-ALL has long faced serious challenges, including limited tolerability, inadequate remission depth and high relapse rates.

Traditional chemotherapy drugs lack the ability to distinguish between rapidly proliferating normal hematopoietic cells and leukemia cells. This not only causes severe and prolonged bone marrow suppression but also makes it difficult to effectively eliminate residual leukemia cells that are in a quiescent or low-proliferative state. In contrast, immunotherapy achieves precise elimination of leukemia cells by specifically targeting leukemia-associated antigens to induce cytotoxic effects. Blina, a bispecific antibody, functions by bridging CD3 on T cells and CD19 on malignant B cells. It activates T cells to directly eliminate CD19-expressing tumor cells (15). InO is a CD22-directed antibody-drug conjugate (ADC). Upon binding to the CD22 antigen on tumor cells, it is internalized and delivers a potent cytotoxic payload, thereby inducing cell death (16). Both agents can effectively eliminate residual leukemia cells following chemotherapy through immune-mediated mechanisms. This property overcomes the biological limitations of traditional chemotherapy in terms of MRD eradication and provides a crucial theoretical basis for improving the depth of remission in first-line treatment.

In recent years, there has been growing interest in the strategy of moving immunotherapy to the front line of treatment. Preliminary studies by Bassan R, Lu J, Jabbour E et al. indicate favorable outcomes with Blina in first-line therapy. These studies report CR rates of 89–100% and MRD negativity rates of 85–94% (17–19). In induction therapy for newly diagnosed elderly B-ALL patients, the addition of InO has also demonstrated encouraging results. Several studies have reported that the use of InO during the induction phase achieves CR rates of 85–89% and MRD negativity rates of 80–96% in elderly patients (20–22). Several clinical trials have demonstrated that the application of Blina/InO in first-line treatment reveals favorable efficacy and safety, but these findings are constrained by the limitation of single-arm trial design. Real-world data on these two drugs are limited. More importantly, existing data primarily focus on elderly patients. The efficacy and safety of Blina/InO in combination with low-dose chemotherapy for first-line induction therapy in fit patients newly diagnosed with B-ALL has not yet been fully established.

In summary, the development of first-line treatment strategies that offer both deeper remission and better tolerability represents an urgent unmet need in young adult B-ALL. Given the significant gap in current evidence regarding young patients, a systematic evaluation of the clinical value of immunotherapy-based strategies in young adult patients newly diagnosed with B-ALL is essential for optimizing first-line treatment strategies, reducing treatment-related toxicity and improving long-term prognosis.

## 2 Materials and methods

### 2.1 Study design and data collection

This retrospective cohort study analyzed medical records of inpatients treated at Qilu Hospital from January 2019 to June 2025. This research protocol has been approved by the Ethics Committee of Qilu Hospital, Shandong University. Baseline clinical data and laboratory test results were obtained from the electronic medical records. Philadelphia chromosome status was also retrospectively assessed. Three experts independently reviewed each patient’s medical records and extracted study characteristics. Discrepancies were resolved through discussion until consensus was reached.

### 2.2 Inclusion criteria

Inclusion criteria: (1) Age 14–60 years; (2) Diagnosis of B-ALL confirmed by morphological, immunological, cytogenetic and molecular (MICM) methods according to the criteria established by the Chinese Medical Association (23), with no prior history of B-ALL; (3) Received first-line induction therapy based on Blina/InO or standard chemotherapy; (4) Availability of complete baseline clinical, treatment and outcome data.

The participants of this study were young patients. Patients aged 14 to 60 years were selected because they typically receive similar treatment regimens at our hospital. Patients with B-ALL arising from chronic myeloid leukemia were excluded. These cases were identified based on a history of chronic myeloid leukemia, clinical findings and laboratory findings, including bone marrow morphology, immunophenotyping, cytogenetic analysis and fluorescence *in situ* hybridization. B-ALL arising from CML differs from primary B-ALL in terms of disease biology, treatment strategies and prognosis. Therefore, excluding these patients helps avoid introducing additional clinical heterogeneity.

### 2.3 Treatment plan

The IG included patients who received Blina/InO as part of their first-line treatment, while the CG included patients who received only conventional chemotherapy as first-line treatment. Blina was given at 9 μg/day from D1 to D7 and then 28 μg/day for the remaining days (D8-D14 or D8-D28). The dosing regimen for InO consisted of 0.8 mg/m^2^ once on D1 and 0.5 mg/m^2^ once each on D8 and D15. Low-intensity chemotherapy regimens included VP (V: Vincristine 1.4 mg/m^2^, ivdrip, D1, D8, D15; P: Prednisone 40 mg/m^2^, po, D1-D5, D8-D12) or VDP (V: Vincristine 1.4 mg/m^2^, ivdrip, D1; D: Daunorubicin 30 mg/m^2^ or Idarubicin 6 mg/m^2^, D1; P: Prednisone 40 mg/m^2^, po, D1-D7). Standard chemotherapy regimens included VDLP, VDCP or VDCLP (V: Vincristine 1.4 mg/m^2^, ivdrip, D1, D8, D15, D22; D: Daunorubicin 30 mg/m^2^ or Idarubicin 6 mg/m^2^, D1-D3; C: Cyclophosphamide 600 mg/m^2^, ivdrip, D1, D15; P: Prednisone 40 mg/m^2^, po, D1-D28; L: Pegaspargase, 2,500 IU/m^2^, im, q2w) (Table 1). Both groups of patients received CNS prophylaxis with intrathecal methotrexate and cytarabine.

**Table 1:** Therapeutic regimens.

Group	Ph,n	Therapeutic regimen, *n*	The treatment course of Blina/InO (d)
IG (*n* = 25)	Negative (*n* = 17)	Blina (*n* = 1) Blina+VP (*n* = 2) Blina+VDP (*n* = 4) InO + VP (*n* = 10)	28 (D1-D28) 28 (D1-D28) 14 (D1-D14) 3 (D1, D8, D15)
	Positive (*n* = 8)	Blina+TKI (*n* = 1) Blina+VP + TKI (*n* = 4) Blina+VDP + TKI (*n* = 2) InO + VP + TKI (*n* = 1)	28 (D1-D28) 28 (D1-D28) 14 (D1-D14) 3 (D1, D8, D15)
CG (*n* = 25)	Negative (*n* = 17)	VDLP (*n* = 8) VDCP (*n* = 1) VDCLP (*n* = 8)	
	Positive (*n* = 8)	VP + TKI (*n* = 1) VDCP+TKI (*n* = 7)	

IG, immunotherapy group; CG, chemotherapy group; Ph, Philadelphia chromosome; Blina, blinatumomab; InO, InOtuzumab ozogamicin; TKI, Tyrosine kinase inhibitor.

### 2.4 Propensity score matching

To minimize the impact of potential confounding factors and selection bias, PSM was applied to the IG and CG to balance baseline characteristics between the two groups. Variables in the propensity score model included age, sex, risk stratification, comorbidities and Philadelphia chromosome status. We performed 1:1 propensity score matching without replacement, applying the nearest-neighbor method with a caliper set at 0.2. After matching, most standardized mean difference (SMD) values were less than 0.1, indicating that good balance was achieved across most covariates. Due to the small sample size in the IG, the matching process prioritized Philadelphia chromosome status.

### 2.5 Outcomes

Primary research objectives included remission rates and MRD negativity rate. Secondary objectives included adverse events.

Composite complete response (cCR) rate was defined as the composite of CR and complete response with incomplete hematologic recovery (CRi). The criteria for CR required < 5% marrow blasts, no evidence of extramedullary disease and peripheral blood count recovery (absolute neutrophil count ≥ 1 × 10^9^/L and absolute platelet count ≥ 100 × 10^9^/L). CRi was the same as CR, but the absolute neutrophil count < 1 × 10^9^/L and/or platelet count < 100 × 10^9^/*L. major* Molecular Remission (MMR) was defined as BCR-ABL transcript level measured by reverse transcription quantitative PCR (RT-qPCR) ≤ 0.1%. Deep Molecular Response (DMR) was defined as BCR-ABL transcript level measured by RT-qPCR ≤ 0.01%.

Following completion of the first cycle of induction therapy, MRD was assessed using multiparametric flow cytometry. An anticoagulated bone marrow sample was collected and centrifuged to isolate mononuclear cells. Fluorescein-labeled monoclonal antibodies and flow cytometry were used to detect cell antigen expression, cell size and the number of intracellular granules, thereby distinguishing between normal and abnormal cells. The instrument used was a 10-color, 3-laser Navios flow cytometer manufactured by Beckman (USA). Data analysis was performed using Kaluza software. MRD negativity was defined as the absence of detectable residual disease assessed by multicolor flow cytometry (MFC) with a sensitivity ≤ 0.01%.

Non-hematologic adverse events included pulmonary infection, septicemia, elevated ALT or AST and elevated creatinine. Hematologic adverse events were assessed using the following indicators: median minimum of neutrophil count, median minimum of hemoglobin level, median minimum of platelet count, duration of neutrophil count < 0.5 × 10^9^/L, duration of hemoglobin level < 80 g/L, duration of platelet count < 30 × 10^9^/L. The numbers of red blood cell transfusions and platelet transfusions were recorded.

### 2.6 Statistical analysis

Continuous data were expressed as mean ± standard deviation (SD) or median (range). If the data were normally distributed, the difference between the two groups was determined using Student’s t-test. If the data were not normally distributed, the Mann–Whitney U test was used. The chi-square test or Fisher’s exact test was used to compare the proportions of cCR, CR, MRD, MMR and DMR. A multivariate logistic regression model was employed to assess factors influencing treatment response in the pre-PSM cohort, with results expressed as odds ratio (OR) and 95% confidence interval (CI). A *p*-value < 0.050 was considered statistically significant. Data management and statistical analysis were performed using SPSS 25.0 and R 4.4.2.

## 3 Results

### 3.1 Patient characteristics

We collected medical records from 223 newly diagnosed B-ALL patients. Ninety patients were excluded, including 89 with missing data and 1 with B-ALL evolved from chronic myeloid leukemia. Eighty-nine patients were excluded due to incomplete baseline data and missing outcome variables.

**Figure 1:**
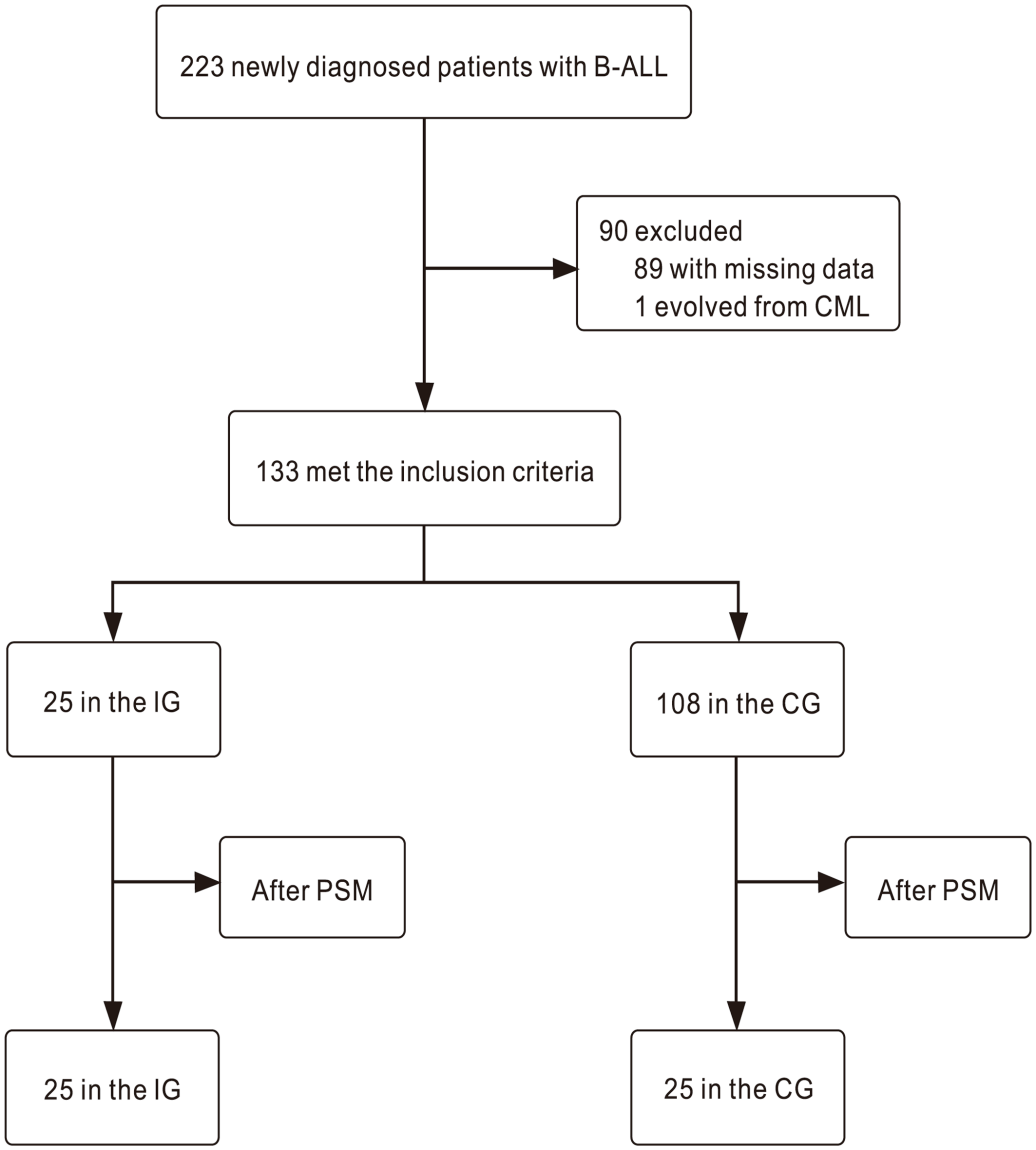
Flow diagram of study design. B-ALL, B-cell acute lymphoblastic leukemia; CML, chronic myeloid leukemia; PSM, propensity score matching.

Ultimately, 133 patients were enrolled in the study and divided into two groups according to their first-line treatment regimens. The IG comprised 25 patients, while the CG included 108 patients. Subsequently, there were 25 patients in each group after PSM (Figure 1).

After matching, baseline characteristics, comorbidities and Philadelphia chromosome status were balanced between the two groups (*p* > 0.050; Table 2). The median (range) age in the IG was 39 (15–58) years, while that in the CG was 39 (15–55) years. Each group included 12 male patients each (48%) and 2 high-risk patients (8%). There were 3 diabetic patients in each group. Three cases of pulmonary infection occurred in the IG (12%), while two occurred in the CG (8%). Each group included 8 Philadelphia chromosome-positive (Ph^+^) patients. All Ph^+^ patients received concurrent treatment with a tyrosine kinase inhibitor (TKI).

**Table 2:** Patient characteristics.

Characteristic	IG (*n* = 25)	CG (*n* = 25)	*P*	SMD
Median age (range), years	39 (15–58)	39 (15–55)	0.793	0
Gender				0
Male, *n* (%)	12 (48%)	12 (48%)	1.000	
Female, *n* (%)	13 (52%)	13 (52%)		
High-risk, *n* (%)	2 (8%)	2 (8%)	1.000	0
Comorbidity				
Type 2 diabetic, *n* (%)	3 (12%)	3 (12%)	1.000	0
Pulmonaryinfection, *n* (%)	3 (12%)	2 (8%)	1.000	0.134
Ph				0
Positive, *n* (%)	8 (32%)	8 (32%)	1.000	
Negative, *n* (%)	17 (68%)	17 (68%)		

IG, immunotherapy group; CG, chemotherapy group; Ph, Philadelphia chromosome.

### 3.2 Treatment efficacy

Both groups of patients achieved cCR. In addition, 22 patients (88%) achieved MRD negativity in the IG, while only 12 patients (48%) did so in the CG. The MRD negativity rate differed significantly between the two groups (88% vs. 48%, *p* = 0.002). With regard to molecular remission, although no statistical significance was observed in MMR (50% vs. 25%) and DMR (50% vs. 12.5%), the proportions were numerically higher in the IG (Table 3). This finding may be limited by the small sample size and requires verification in larger cohorts.

**Table 3:** Comparison of treatment efficacy.

Efficacy outcome	IG (*n* = 25)	CG (*n* = 25)	*P*
cCR, *n* (%)	25 (100%)	25 (100%)	NR
CR, *n* (%)	24 (96%)	23 (92%)	1.000
MRD negativity (MFC), *n* (%)	22 (88%)	12 (48%)	0.002

Ph^+^ subgroup	IG (*n* = 8)	CG (*n* = 8)	*P*
MMR (BCR-ABL), *n* (%)	4 (50%)	2 (25%)	0.608
DMR (BCR-ABL), *n* (%)	4 (50%)	1 (12.5%)	0.282

IG, immunotherapy group; CG, chemotherapy group; NR, not reported; cCR, composite complete remission, include complete remission and complete remission with incomplete hematologic recovery; CR, complete remission; MRD, minimal residual disease; Ph^+^, Philadelphia chromosome positive; MMR, major molecular remission; DMR, deep molecular response.

### 3.3 Relation between baseline characteristics and MRD

Multivariate logistic regression analysis was employed to determine the relationship between baseline characteristics and MRD negativity. The results showed that immunotherapy remained associated with MRD negativity after one cycle of induction therapy. Other factors, such as age, sex, high-risk stratification, Philadelphia chromosome status, comorbidities and TKI treatment were not associated with MRD negativity in patients (all *p* > 0.050; Table 4).

**Table 4:** Multivariate logistic regression analysis of factors influencing MRD negativity.

Factor	Pre-PSM (*n* = 133)		
	OR	95%CI	*P*
Age	0.984	0.955–1.014	0.304
Sex	0.472	0.220–1.014	0.054
High-risk	0.775	0.127–4.728	0.783
Ph	0.699	0.196–2.409	0.581
Comorbidity			
Type 2 diabetic	0.586	0.109–3.135	0.532
Pulmonary infection	1.453	0.371–5.687	0.591
Immunotherapy	9.820	2.585–37.299	0.001
Targeted therapy			
TKI	1.422	0.384–5.263	0.598

Ph, Philadelphia chromosome; MRD, minimal residual disease.

TKI, tyrosine kinase inhibitor.

### 3.4 Safety

Regarding hematologic toxicity, the IG had a higher median minimum of neutrophil count (0.14 vs. 0.01 × 10^9^/L, *p* < 0.001) and a higher median minimum of platelet count (37 vs. 8 × 10^9^/L, *p* = 0.001) compared with the CG. No significant difference was found between groups regarding the median lowest hemoglobin level (59 vs. 53 g/L, *p* = 0.066). Furthermore, the IG experienced shorter duration of neutrophil count < 0.5 × 10^9^/L (3 vs. 11 d, *p* = 0.033), hemoglobin level < 80 g/L (11 vs. 20 d, *p* = 0.038) and platelet count < 30 × 10^9^/L (0 vs. 8 d, *p* = 0.006). Additionally, regarding non-hematologic adverse events, fewer patients in the IG experienced pulmonary infections (44% vs. 84%, *p* = 0.003). There was no significant difference in the incidence of sepsis and elevated liver enzymes (8% vs. 16%, *p* = 0.663; 40% vs. 40%, *p* = 1.000) between the two groups. No renal dysfunction was noted in either group of patients. Regarding blood product transfusions, the IG required significantly fewer red blood cell and platelet transfusions (4 vs. 8 u, *p* = 0.012; 0 vs. 48 u, *p* < 0.001) (Table 5).

**Table 5:** Comparison of safety.

Adverse event	IG (*n* = 25)	CG (*n* = 25)	*P*
Hematologic adverse event, M (range)			
Min of NEU count (×10^9^/L)	0.14 (0.01–1.99)	0.01 (0–0.74)	< 0.001
Duration of NEU count <0.5 × 10^9^/L, d	3 (0–20)	11 (0–26)	0.033
Min of HB (g/L)	59 (37–103)	53 (38–89)	0.066
Duration of HB < 80 g/L,d	11 (0–40)	20 (0–27)	0.038
Min of PLT count (×10^9^/L)	37 (5–210)	8 (1–137)	0.001
Duration of PLT count <30 × 10^9^/L, d	0 (0–34)	8 (0–25)	0.006
Non-hematological adverse event, *n* (%)			
Pulmonary infection	11 (44%)	21 (84%)	0.003
Septicemia	2 (8%)	4 (16%)	0.663
Elevated ALT or AST	10 (40%)	10 (40%)	1.000
Elevated creatinine	0 (0%)	0 (0%)	NR
Volume of blood transfused, M (range)			
RBC, u	4 (0–23)	8 (0–18)	0.012
PLT, u	0 (0–288)	48 (0–304)	< 0.001

IG, immunotherapy group; CG, chemotherapy group; M, median; Min, minimum; NEU, neutrophil; HB, hemoglobin; PLT, platelet; ALT, alanine aminotransferase; AST, aspartate aminotransferase; NR, not reported; RBC, red blood cell.

### 3.5 Subgroup analysis

Additionally, a subgroup analysis was conducted between the Blina and InO groups. The baseline characteristics of the two groups were largely comparable, except for the proportion of Ph^+^ patients (Supplementary Table S1). Further comparisons of efficacy and safety outcomes between the two groups revealed no statistically significant differences (Supplementary Table S2).

## 4 Discussion

This real-world study aimed to evaluate the efficacy and safety of adding Blina/InO to first-line treatment in newly diagnosed B-ALL patients who were eligible for intensive chemotherapy. In the study, the CG attained a CR rate of 92% and an MRD negativity rate of 48% following one cycle of induction therapy, which was consistent with existing data (24, 25).

In this retrospective study, first-line treatment based on immunotherapy was associated with encouraging early treatment responses in newly diagnosed B-ALL patients eligible for intensive chemotherapy. The study indicates that in the assessment after the first cycle of induction therapy, the MRD negativity rate in the IG was markedly higher than that in the CG (88% vs. 48%, *p* = 0.002). Our findings are consistent with those of other large collaborative studies evaluating the efficacy of incorporating Blina/InO into first-line chemotherapy regimens for elderly patients. For example, in the GMALL Bold trial, Goekbuget N et al. demonstrated that older patients receiving one cycle of induction therapy including low-intensity chemotherapy and Blina achieved an 82% MRD negativity rate, consistent with the results observed in our IG (26). Furthermore, in a Phase 2 trial report from MD Anderson Cancer Center, the 84% MRD negativity rate observed in elderly patients following reduced-dose chemotherapy and InO closely aligns with the 88% MRD negativity rate observed in our younger cohort (19). Importantly, multivariate logistic regression analysis further revealed that immunotherapy was associated with MRD negativity, while baseline characteristics, comorbidities and other concurrent therapies were unrelated to treatment response. These results further suggested that first-line regimens based on immunotherapy may be associated with early MRD negativity in young patients.

In terms of safety, first-line treatment regimens based on immunotherapy showed better tolerability to hematologic toxicity. Patients in the IG experienced milder myelosuppression, with higher median lowest neutrophil count (*p* < 0.001) and median lowest platelet count (*p* = 0.001) compared with the CG. Furthermore, the bone marrow suppression in the IG recovered more rapidly. The median time to recovery of neutrophil count (*p* = 0.033) and platelet count (*p* = 0.006) was observably shorter than that in the CG and that reported by Lu et al. (25). The incidence of pulmonary infection in the IG was markedly lower than that in the CG (44% vs. 84%, *p* = 0.003). Notably, the IG required fewer blood product transfusions (Red blood cell, *p* = 0.012; Platelet, *p* < 0.001).

The study focused on young adult patients newly diagnosed with B-ALL receiving first-line immunotherapy, for whom real-world evidence regarding first-line immunotherapy strategies remains limited. Moreover, this study employed PSM to control for potential confounding factors and reduce baseline imbalances between groups. In addition, this study evaluated clinically meaningful outcome measures, including remission response, MRD negativity and adverse events. The results suggested that an immunotherapy-based first-line treatment strategy may offer potential advantages.

Some encouraging results were achieved, but our research has several limitations. The PSM method could only adjust for measured covariates but could not control for unmeasured confounders, thereby leaving residual bias inevitable. Furthermore, this study primarily focused on early efficacy and safety following induction therapy. Due to limitations related to follow-up duration, the number of events, post-treatment strategies and transplantation, no survival analysis was conducted. Lastly, due to limited data capture in this retrospective study, the assessment of adverse events was constrained, including the absence of CTCAE grading and immunotherapy-specific toxicities.

## 5 Conclusion

In summary, compared with standard chemotherapy regimens, first-line immunotherapy strategies incorporating Blina/InO showed preliminary encouraging signs of early efficacy. Moreover, immunotherapy-based first-line treatment may be associated with milder bone marrow suppression and lower blood transfusion requirements. However, these findings are still in the exploratory stage and intended to serve as supplementary real-world evidence to inform future research directions. Therefore, large-scale prospective studies are needed to validate these preliminary findings and further clarify the clinical value of first-line immunotherapy regimens.

## Data Availability

The original contributions presented in the study are included in the article/Supplementary material, further inquiries can be directed to the corresponding author/s.
