# Supplementary material for: Immunotherapy with or without low-intensity chemotherapy versus conventional chemotherapy as first-line treatment for newly diagnosed B-ALL patients fit for intensive chemotherapy: a propensity score-matched study
Source: Front Med (Lausanne). 2026 Jun 18;13:1843800. doi: 10.3389/fmed.2026.1843800 (PMC13322836; doi:10.3389/fmed.2026.1843800)
Supplement: Supplementary file 1 [file Data_Sheet_1.PDF]

Supplementary Table 1 Patient Characteristics

**Characteristic**	**Blina(n = 14)**	**InO(n = 11)**	**P**
Age,M±SD,years	37.29±16.23	35.82±15.43	0.821
Sex			
Male,n(%)	6(42.9%)	6(54.5%)	0.695
Female,n(%)	8(57.1%)	5(45.5%)	
High-risk,n(%)	1(7.1%)	0(0.0%)	1.000
Ph			
Positive,n(%)	7(50.0%)	1(9.1%)	0.042
Negative,n(%)	7(50.0%)	10(90.9%)	
Comorbidity			
Type 2 diabetic,n(%)	2(14.3%)	1(9.1%)	1.000
Pulmonary infection,n(%)	3(21.4%)	0(0.0%)	1.000

Abbreviations: Blina,Blinatumomab;InO,Inotuzumabm Ozogamicin;M,mean; SD,standard deviation;Ph,Philadelphia chromosome.

Supplementary Table 2 Efficacy and safety

**Efficacy outcome**	**Blina(n =14)**	**InO(n = 11)**	**P**
cCR, n(%)	14(100%)	11(100%)	NR
CR, n(%)	13(92.9%)	11(100%)	1.000
MRD negativity(MFC), n(%)	12(85.7%)	10(90.9%)	1.000
**Ph^+ subgroup**	**Blina (n = 7)**	**InO(n = 1)**	
MMR(BCR-ABL), n(%)	4(57.1%)	0(0%)	1.000
DMR(BCR-ABL), n(%)	4(57.1%)	0(0%)	1.000
**Adverse event**	**Blina(n = 14)**	**InO(n = 11)**	
Hematologic adverse event, M(range)			
Min of NEU count ($\times10^9$/L)	0.15(0.01-0.53)	0.14(0.01-1.99)	1.000
Duration of NEU count＜0.5×10^9/L,d	5(0-20)	3(0-18)	0.761
Min of HB (g/L)	58(37-103)	59(41-102)	0.913
Duration of HB＜80g/L,d	11(0-40)	11(0-24)	0.848
Min of PLT count($\times10^9$/L)	35(5-210)	37(5-109)	0.935
Duration of PLT count＜30×10^9/L,d	8(0-40)	4(0-21)	0.759
Non-hematologicaladverse event, n(%)			
Pulmonary infection	8(57.1%)	3(27.3%)	0.227
Septicemia	1(7.1%)	1(9.1%)	1.000
Elevated ALT or AST	7(50%)	3(27.3%)	0.414
Elevated creatinine	0(0.0%)	0(0.0%)	NR
Volume of blood transfused, M(range)			
RBC, u	3(0-23)	4(0-12)	0.561
PLT, u	16(0-288)	0(0-80)	0.174

Abbreviations: cCR, composite complete remission, include complete remission and

complete remission with incomplete hematologic recovery;CR, complete remission;MRD, minimal residual disease;Blina,Blinatumomab;InO,Inotuzumabm Ozogamicin;MMR, major molecular remission;DMR, deep molecular response;M, median;Min,minimum;NEU, neutro phil;HB, hemoglobin;PLT, platelet; ALT, alanineaminotransferase; AST,aspartate aminotransfe rase; NR, not reported; RBC, red blood cell.
